# HELPING BEHAVIORS AND COGNITIVE FUNCTIONING: IMPLICATIONS FOR INDIVIDUALS WITH COGNITIVE IMPAIRMENT AND DEMENTIA

**DOI:** 10.1093/geroni/igae098.4205

**Published:** 2024-12-31

**Authors:** Shiyang Zhang, HaeJin Jang, Sae Hwang Han

**Affiliations:** University of Texas at Austin, Austin, Texas, United States; University of Texas at Austin, Austin, Texas, United States; University of Texas at Austin, Austin, Texas, United States

## Abstract

Formal volunteering is widely recognized for its health benefits, including improved cognitive functioning and delayed onset of cognitive impairment and dementia. However, the extent to which these cognitive benefits vary across individuals with different levels of cognitive functioning remains unclear, and the impact of informal helping (e.g., helping friends, neighbors) on cognition is even less well understood. To address these gaps, we analyze data from the U.S. Health and Retirement Study (1998–2020; 199,126 observations from 35,004 individuals) using unconditional quantile regression models. This allowed for investigating the heterogeneous effects of two forms of helping behaviors–formal volunteering and informal helping–on cognitive functioning, measured with the Telephone Interview for Cognitive Status (TICS; range 0–27), across the cognitive functioning distribution. Our findings reveal that the cognitive benefits of both helping behaviors become progressively more pronounced for individuals experiencing worse cognitive functioning, including those with cognitive impairment (TICS score: 7–11) and dementia (score: 0–6). These unique results challenge the prevailing assumption that healthier individuals are more likely to engage in helping behaviors and thus accrue more benefits. Instead, this study highlights that helping behaviors may be particularly beneficial for individuals at the lower end of the cognitive functioning distribution, and shed a positive light on many older adults who continue to help others despite having health challenges. Our findings support recommendations for healthcare practitioners to devise and implement innovative strategies that integrate helping behaviors as part of healthcare to promote cognitive health and well-being among older adults, especially those at risk of cognitive impairment.

